# Interactive effects of sleep and physical activity on depression among rural university students in China

**DOI:** 10.3389/fpsyg.2023.1240856

**Published:** 2023-09-28

**Authors:** Yang Yang, Zhen Zhang, Jia Liu, Hongmin Cao

**Affiliations:** ^1^Physical Education Institute, Huanghuai University, Zhumadian, China; ^2^School of Physical Education, Chizhou University, Chizhou, China

**Keywords:** sleep quality, MVPA, rural areas, university students, interaction effects

## Abstract

**Background:**

Decreased sleep quality and physical activity among university students have become important concerns, while depressive symptoms are on the rise, especially in rural areas. Previous studies have confirmed the association between sleep quality and physical activity and depressive symptoms, but the effect of their interaction on depressive symptoms has been less studied. For this reason, the present study analyzed the interaction between sleep quality and physical activity on depressive symptoms to provide reference and assistance for mental health promotion and intervention for university students in rural areas of China.

**Methods:**

In this study, we investigated sleep quality, physical activity, and depressive symptoms in 11,423 university students in rural areas of China. The association of sleep quality and physical activity with depressive symptoms was analyzed by using univariate analysis and logistic regression analysis. And further analyzed the association between the interaction effect of sleep quality and physical activity and depressive symptoms.

**Results:**

The proportion of university students with depressive symptoms in rural China was 33.3%, with a higher proportion of girls students (36.7%) having depressive symptoms than boys students (28.8%). Logistic regression analysis of the interaction between sleep quality and physical activity and depressive symptoms in university students showed that university students with poor sleep quality and MVPA>60 min/d (OR = 4.40,95% CI: 3.75–5.05) had the highest risk of depressive symptoms (*p* < 0.001). University students with moderate sleep quality and MVPA of 30–60 min/d (OR = 1.18, 95% CI: 0.75–1.61) had the lowest risk of depressive symptoms (*p* < 0.001). Moreover, there was no gender difference in this result.

**Conclusion:**

The prevalence of depressive symptoms was higher among university students in rural areas of China. There was an interaction between sleep quality and MVPA on depressive symptoms, maintaining a good quality of sleep and an appropriate length of MVPA has a contributory effect on mental health, and the same trend was found for both boys and girls students. It is recommended that university students in rural areas of China should first maintain proper sleep quality while maintaining 30–60 min of MVPA per day, which may reduce the risk of depressive symptoms.

## 1. Introduction

Several studies have confirmed that adolescent sleep deprivation problems may be an important risk factor for mental health problems (Feng et al., 2014; Becker et al., 2018). One study confirmed that sleep-deprived adolescents have an increased risk of depressive symptoms and anxiety compared to those who get enough sleep (Zou et al., 2020). It has also been shown that adolescent boys who slept less than 8.5 h/day and girls who slept less than 7.5 h/day had significantly higher scores on depressive symptoms (Ojio et al., 2016). A study of twin children also confirmed that sleep duration and sleep quality were significantly associated with mental health scores after excluding genetic factors (Matamura et al., 2014). Similarly, a study of university students confirmed a significant association between university students’ sleep quality and an increased risk of developing depressive symptoms (Kim et al., 2022). However, with the increasing employment pressure and academic pressure of university students, the problem of poor sleep quality of university students has become more and more prominent, especially the problem of sleep deprivation of girls students is significantly higher than that of boys students (Yang et al., 2022). A survey revealed that 51% of university students in India have varying degrees of sleep quality problems and have serious negative effects on university students’ living and learning, and poorer sleep quality university students are associated with depressive symptoms (OR = 1.58) and anxiety (OR = 1.38) showed a significant positive correlation (Ghrouz et al., 2019). Similarly, a study of university students in China confirmed that 71% of university students in China have poor sleep quality, with 14% of them sleeping less than 6 h, and are at increased risk of mental health problems (Li et al., 2019). A survey report shows that 15.97% of Chinese university students have different degrees of sleep quality problems, and have a certain negative impact on mental health, which needs to attract the attention and concern of the education department, parents and students themselves (Wang et al., 2022).

The effect of physical activity (PA) on adolescent mental health has also been confirmed by several studies, especially the effect of MVPA on mental health is more significant. The study confirmed that there is a significant association between physical activity and depressive symptoms and anxiety in adolescents (Coakley et al., 2021). Another meta-analysis also showed that an increase in the duration of moderate-to-high intensity physical activity was associated with a decrease in depressive symptoms scores, suggesting a better reduction in depressive symptoms levels and a positive effect on mental health (Radovic et al., 2017). A study of Chinese adolescents confirmed that higher MVPA was associated with a lower risk of depressive symptoms in boys (OR = 0.68, 95% CI: 0.57–0.81), and the same trend was found in girls (OR = 0.67, 95% CI: 0.54–0.83) (Liu et al., 2020). It has also been shown that active physical activity during adolescence and a reasonable level of physical activity has a positive effect on mental health, and that this effect continues into adulthood and has a positive impact on the development of mental health in adulthood (Appelqvist-Schmidlechner et al., 2018). However, the problem of insufficient physical activity among adolescents has become a common concern in countries around the world. A survey reports that 31.1% of adults worldwide lack physical activity, 80.3% of adolescents spend less than 1 hour per day on MVPA, and the problem of lack of physical activity is more pronounced among girls than boys (Hallal et al., 2012). A survey of 2022 Chinese youth physical activity report cards shows that only 35.5% of Chinese youth meet the world-recognized recommended standard of at least 60 min of MVPA per day (Liu et al., 2023). This suggests that the problem of physical inactivity among adolescents deserves attention and concern, and that the negative psychological effects of physical inactivity should also be emphasized. However, it is also important to note whether the negative impact of physical activity on mental health is also influenced by other covariates.

The above studies fully confirm that sleep and physical activity are important factors affecting adolescents’ mental health. Unfortunately, these studies mainly analyzed the effects of sleep or physical activity on adolescents’ mental health problems such as depressive symptoms or anxiety from a single dimension. However, factors affecting mental health are multifaceted, and few previous studies have analyzed the effect of the interaction between sleep quality and physical activity on psychological problems such as depressive symptoms. For example, whether the lack of physical activity has some effect on mental health when adolescents have better sleep quality and vice versa needs to be confirmed. It is also unclear whether there are gender differences in the effect of the interaction between sleep quality and physical activity on depressive symptoms. Although previous studies have confirmed the interaction effect of sleep quality and physical activity on the risk of death in older adults (Bellavia et al., 2014). Additionally, there have been studies within the athlete population (Irandoust et al., 2019; Taheri et al., 2023). However, to the best of our knowledge, no studies have investigated the interaction of sleep quality and physical activity on depressive symptoms in adolescents and no research studies have been conducted on university students in rural China.

China is a developing country with the majority of its population living in rural areas. The mental health of university students in rural areas of China has always been of concern to scholars due to the combined effects of the shortage of educational resources, limited access to mental health resources, high academic pressure, and limited medical and health conditions. Several previous studies have confirmed that the sleep quality, physical activity and mental health of university students in rural areas of China are not optimistic. For example, 41.9% of university students in rural areas have mental health problems, which is higher than the finding of 35.8% in urban areas (Zhang et al., 2017). In addition, the survey also showed that 76.4% of Chinese rural adolescents had sleep quality problems, a higher result than urban adolescents, and girls were more likely to have sleep quality problems than boys (OR = 1.36, *p* = 0.01), and sleep quality problems have become a common health problem among adolescents in rural areas of China (Li et al., 2023). Given the large population of university students in rural areas of China, and the problems in mental health. We conducted a cross-sectional survey of sleep quality, physical activity, and depressive symptoms among university students in rural areas of China. Based on previous studies, the present study hypothesized that there is an interaction between sleep quality and physical activity and psychological symptoms among university students in rural China. This study is the first to examine the interaction effects of sleep quality and physical activity on depressive symptoms among university students in rural China, and to further explore the gender differences. It provides a reference for physical and mental health promotion and intervention for university students.

## 2. Methodology

### 2.1. Participants

**Figure 1:**
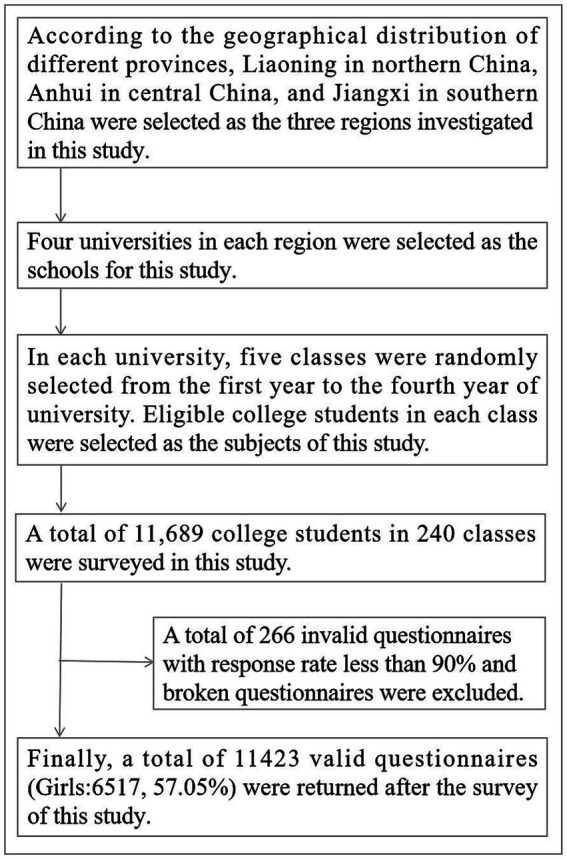
Flow chart of university students sampling in rural areas of China.

A three-stage stratified whole-group sampling method was used for participant sampling in this study. In the first stage, Liaoning in northern China, Anhui in central China, and Jiangxi in southern China were selected as the three regions investigated in this study according to the geographical distribution of different provinces. In the second stage, four universities in each region were selected separately as the schools to be investigated in this study. In the third stage, five teaching classes were selected from each university in each of the first to fourth year of university and the whole group, and the eligible university students in the classes were used as the respondents of this study to conduct the relevant survey. The inclusion criteria for university students in this study were: university students enrolled in school, no major physical or mental illness, and voluntary acceptance of the study. Finally, a total of 11,689 university students in 240 teaching classes were surveyed in this study. 266 invalid questionnaires with response rate not exceeding 90% and broken questionnaires were excluded, and a total of 11,423 valid questionnaires were returned (Girls:6517, 57.05%). Of these, 3,808 (33.34%) questionnaires were from Liaoning, 3,812 (33.37%) from Anhui, and 3,803 (33.29%) from Jiangxi. The specific university students sampling process is shown in Figure 1.

Written informed consent was obtained from the subjects and parents prior to the investigation of this study, and the investigation was conducted on a voluntary basis. This research investigation was approved by the Human Ethics Committee of Huang huai University (201902-0219).

### 2.2. Sleep quality

The assessment of sleep quality was investigated using the internationally recognized pittsburgh sleep quality index (PSQI). The scale consists of 19 entries with 7 factors, namely subjective sleep quality, sleep latency, sleep persistence, habitual sleep efficiency, sleep disturbance, use of hypnotic drugs, and daytime dysfunction. Each factor is scored 0–3 respectively, with a total score range of 0–21. A score of 7 was used as the cut-off standard for sleep disorders. In this study, Sleep quality was classified as Good (≤5 points), Moderate (6–7 points), Poor (>7 points). The Cronbach alpha coefficient of the scale was 0.79 (Buysse et al., 1989).

### 2.3. Physical activity

Participants’ physical activity was surveyed using the international physical activity scale (IPAQ) short form. The scale consists of 7 items. The number and duration of physical activity of different intensities in the past 7 days were investigated to calculate the average daily physical activity duration in the past 7 days. According to the international physical activity recommendation standard, the daily duration of moderate to high intensity physical activity should be at least 60 min as the standard (Sun et al., 2022). In this study, the MVPA time was classified as <30 min/d, 30–60 min/d, and > 60 min/d. The IPAQ has acceptable reliability and validity for Chinese university students (Qu and Li, 2004).

### 2.4. Basic information and covariates

The survey of basic information of the participants mainly included information about the province, school, college, class, and age of the participants. The survey of covariates mainly included father’s education level, mother’s education level, socioeconomic status (SES), and nutritional status. In this study, father’s education level and mother’s education level were divided into three levels: elementary school and below, middle school or high school, and college and above. The socioeconomic status was categorized into low grade (<4,000/month), medium grade (4000-8000/month), and high grade (>8,000/month) based on the average monthly household economic income. Nutritional status was calculated based on the height and weight of the test participants, and the body mass index was calculated as weight (kg)/height (m)^2^, and was classified into Slimmer (BMI < 18.5 kg/m^2^), Normal (18.5 kg/m^2^ < BMI < 24.0 kg/m^2^), Overweight (BMI < 24.0 kg/m^2^), and Obese (BMI < 24.0 kg/m^2^) according to the Chinese adult overweight and obesity classification standard, Overweight (BMI < 28.0 kg/m^2^), and Obese (BMI ≥ 28.0 kg/m^2^). Height and weight were tested according to the Chinese National Student Physical Fitness Test Standard, with height accuracy to 0.1 cm and weight accuracy to 0.1 kg (CNSSCH Association, 2022).

### 2.5. Quality control

Participants were surveyed by subject-trained teachers who served as survey staff. Communication with the school was made before the survey and students were gathered in the classroom for the test and survey. A uniform guideline was used to explain the purpose and requirements of the survey to the participants before the questionnaire was administered. Questionnaires were distributed on the spot and then filled out, and returned on the spot after completion. The questionnaires were filled out using anonymous numbering. The height and weight tests were conducted by dedicated staff to ensure the accuracy of the test. The questionnaires were again verified by the staff after retrieval, and participants were asked to fill in the missing and wrong entries in time. The questionnaire was asked to be filled out independently by college students, without the constraints of time or others. Participants were given material incentives, such as school supplies, for completing the questionnaire. Investigators are more fixed and cannot be replaced at will, in order to guarantee the uniformity of survey standards and ensure the accuracy of survey data.

### 2.6. Statistical analysis

Because of the differences in the detection rates of depressive symptoms in university students boys and girls, our study was analyzed stratified by gender. The basic status, covariates, physical activity, sleep quality and depressive symptoms of different gender university students in rural areas of China were expressed as percentages. Comparisons of the detection rates of depressive symptoms among different sleep quality and physical activity university students were performed by chi-square test. The association of different sleep quality and physical activity university students with depressive symptoms was performed by binary logistic regression analysis after gender stratification. Model 1 was the crude model, and Model 2 was adjusted for age, father’s education, mother’s education, SES, and BMI on the basis of Model 1. The interaction of sleep quality and physical activity on depressive symptoms in university students was analyzed by ordered logistic regression. Age, father’s education, mother’s education, SES, and BMI were adjusted as covariates for the analysis. The odds ratio (OR) and 95% confidence intervals (95% CI) were presented, respectively. *p* < 0.05 was considered a statistically significant difference. Statistical analyzes were processed and plotted using SPSS 25.0 software (IBM, Armonk, NY, United States) and Graph Pad Prism 8.0.2 (Graph Pad Software, Inc., CA).

## 3. Results

In this study, a total of 11,423 university students (boys: 4906, 42.95%) with a mean age of (19.12 ± 1.04) years were surveyed in rural China. Table 1 shows that father’s education level and mother’s education level were the lowest in college and above, with 9.5 and 5.3%, respectively. Regarding Nutritional status, the proportions of slimmer, normal, overweight, and obese were 15.9, 54.1, 12.6, and 17.5%, respectively, and the proportion of slimmer was higher in girls (20.2%) than in boys (10.0%). In terms of sleep quality, the proportions of good, moderate, and poor were 37.0, 27.1, and 35.9%, respectively. The proportion of university students with MVPA <30 min/d was 72.2%, and it was higher in girls (80.1%) than in boys (61.6%). The proportion of those with depressive symptoms was 33.3%, which was also higher in girls students (36.7%) than in boys (28.8%).

**Table 1:** Basic condition, sleep quality, physical activity, depressive symptoms detection rate of university students by gender in rural areas of China.

Items	Boys	Girls	Total
Number	4,906	6,517	11,423
Father’s education level, *N* (%)			
Elementary school and below	1,365 (27.8)	1,644 (25.2)	3,009 (26.3)
Middle school or high school	3,085 (62.9)	4,241 (65.1)	7,326 (64.1)
College and above	456 (9.3)	632 (9.7)	1,088 (9.5)
Mother’s education level, *N* (%)			
Elementary school and below	2,367 (48.2)	2,880 (44.2)	5,247 (45.9)
Middle school or high school	2,255 (46.0)	3,314 (50.9)	5,569 (48.8)
College and above	284 (5.8)	323 (5.0)	607 (5.3)
SES, *N* (%)			
Low grade	867 (17.7)	902 (13.8)	1769 (15.5)
Medium grade	3,385 (69.0)	4,713 (72.3)	8,098 (70.9)
High Grade	654 (13.3)	902(13.8)	1,556 (13.6)
Nutritional status, *N* (%)			
Slimmer	493 (10.0)	1,318 (20.2)	1811 (15.9)
Normal	2,613 (53.3)	3,563 (54.7)	6,176 (54.1)
Overweight	933 (19.0)	503 (7.7)	1,436 (12.6)
Obese	867 (17.7)	1,133 (17.4)	2000 (17.5)
Sleep quality, *N* (%)			
Good	1953 (39.8)	2,270 (34.8)	4,223 (37.0)
Moderate	1,341 (27.3)	1760 (27.0)	3,101 (27.1)
Poor	1,612 (32.9)	2,487 (38.2)	4,099 (35.9)
MVPA (min/d), *N* (%)			
<30	3,022 (61.6)	5,222 (80.1)	8,244 (72.2)
30–60	1,285 (26.2)	967 (14.8)	2,252 (19.7)
>60	599 (12.2)	328 (5.0)	927 (8.1)
SDS, *N* (%)			
No	3,493 (71.2)	4,128 (63.3)	7,621 (66.7)
Yes	1,413 (28.8)	2,389 (36.7)	3,802 (33.3)

SES, socioeconomic status; MVPA, Moderate-to-Vigorous Physical Activity; SDS, self-rating depression scale.

Table 2 shows that there is a significant difference in the detection rate of depressive symptoms by sleep quality and MVPA among boys and girls and overall among university students in rural areas of China (*p* < 0.001). Overall, the detection rate of depressive symptoms was highest in students with poor sleep quality (52.55%) and lowest in university students with good sleep quality (20.18%). The difference was statistically significant (*χ*^2^ value of 1092.902, *p* < 0.001). The detection rate of depressive symptoms was lowest in university students with MVPA >60 min/d (10.90%) and highest in university students with MVPA <30 min/d (38.73%). The difference was statistically significant (*χ*^2^ value was 436.094, *p* < 0.001).

**Table 2:** Comparison of the detection rates of different sleep quality and physical activity and depressive symptoms among university students in rural areas of China.

Gender	Category	Group	Number of people	SDS		
				*N* (%)	*χ*^2^ value	*p* value
Boys	Sleep quality	Good	1953	291 (14.90)	595.412	<0.001
		Moderate	1,341	301 (22.45)		
		Poor	1,612	821 (50.93)		
	MVPA (min/d)	<30	3,022	989 (32.73)	120.672	<0.001
		30–60	1,285	361 (28.09)		
		>60	599	63 (10.52)		
Girls	Sleep quality	Good	2,270	561 (24.71)	502.045	<0.001
		Moderate	1760	495 (28.13)		
		Poor	2,487	1,333 (53.6)		
	MVPA (min/d)	<30	5,222	2,204(42.21)	349.749	<0.001
		30–60	967	147 (15.20)		
		>60	328	38 (11.59)		
Total	Sleep quality	Good	4,223	852 (20.18)	1092.902	<0.001
		Moderate	3,101	796 (25.67)		
		Poor	4,099	2,154 (52.55)		
	MVPA (min/d)	<30	8,244	3,193 (38.73)	436.094	<0.001
		30–60	2,252	508 (22.56)		
		>60	927	101 (10.90)		

MVPA, Moderate-to-Vigorous Physical Activity; SDS, self-rating depression scale.

Overall, with the group of university students with good sleep quality as a reference, after adjusting for relevant influences. Model 2 shows that university the risk of depressive symptoms was higher in university students with poor sleep quality (OR = 4.37, 95% CI: 3.97 ~ 4.82) (*p* < 0.001). In terms of MVPA, university students with MVPA 30-60 min/d (OR = 2.32, 95% CI: 1.84 ~ 2.92) had an increased risk of depressive symptoms compared to university students with MVPA >60 min/d as the reference group. University students with MVPA <30 min/d (*OR* = 5.05, 95% CI: 4.09 ~ 6.24) had the highest risk of developing depressive symptoms (*p* < 0.001). The results of logistic regression analysis for boys and girls are shown in Table 3.

**Table 3:** Logistic regression analysis of different sleep quality and physical activity and depressive symptoms among university students in rural areas of China.

Gender	Variable	Group	Model 1		Model 2	
			OR (95% CI)	*p-*value	OR (95% CI)	*p-*value
Boys	Sleep quality	Good	1.00 (Reference group)		1.00 (Reference group)	
		Moderate	1.65 (1.38 ~ 1.98)	<0.001	1.65 (1.38 ~ 1.97)	<0.001
		Poor	5.93 (5.06 ~ 6.94)	<0.001	6.02 (5.14 ~ 7.07)	<0.001
	MVPA (min/d)	>60	1.00 (Reference group)		1.00 (Reference group)	
		30–60	3.32 (2.49 ~ 4.43)	<0.001	3.26 (2.44 ~ 4.35)	<0.001
		<30	4.14 (3.15 ~ 5.43)	<0.001	4.12 (3.14 ~ 5.41)	<0.001
Girls	Sleep quality	Good	1.00 (Reference group)		1.00 (Reference group)	
		Moderate	1.19 (1.04 ~ 1.37)	0.015	1.21 (1.05 ~ 1.40)	0.008
		Poor	3.52 (3.11 ~ 3.98)	<0.001	3.50(3.09 ~ 3.97)	<0.001
	MVPA (min/d)	>60	1.00 (Reference group)		1.00 (Reference group)	
		30–60	1.37 (0.94 ~ 2.00)	0.107	1.34 (0.91 ~ 1.96)	0.136
		<30	5.57 (3.96 ~ 7.85)	<0.001	5.43 (3.85 ~ 7.67)	<0.001
Total	Sleep quality	Good	1.00 (Reference group)		1.00 (Reference group)	
		Moderate	1.37 (1.22 ~ 1.53)	<0.001	1.38 (1.24 ~ 1.55)	<0.001
		Poor	4.38 (3.98 ~ 4.83)	<0.001	4.37 (3.97 ~ 4.82)	<0.001
	MVPA (min/d)	>60	1.00 (Reference group)		1.00 (Reference group)	
		30–60	2.38 (1.90 ~ 3.00)	<0.001	2.32 (1.84 ~ 2.92)	<0.001
		<30	5.17 (4.19 ~ 6.39)	<0.001	5.05 (4.09 ~ 6.24)	<0.001

Model 1 is the crude model, model 2 is based on model 1 with adjustment of age, father’s education, mother’s education, SES, and BMI. Sleep quality: Good is ≤ 5, Moderate is 6–7, Poor is > 7. OR, odds ratio; 95% CI, confidence intervals. MVPA, Moderate-to-Vigorous Physical Activity.

Overall, it can be seen that university students with good sleep quality and MVPA >60 min/d as the reference group, university students with poor sleep quality and MVPA >60 min/d (OR = 4.40,95% CI: 3.75 ~ 5.05) had the highest risk of developing depressive symptoms (*p* < 0.001). University students with moderate sleep quality and MVPA of 30 ~ 60 min/d (OR = 1.18, 95% CI: 0.75 ~ 1.61) had the lowest risk of developing depressive symptoms (*p* < 0.001). Thus, the interaction between university students’ sleep quality and MVPA and depressive symptoms was observed. University students should maintain proper sleep quality and also maintain 30–60 min of MVPA per day to reduce the interaction between university students’ sleep quality and MVPA and depressive symptoms was found. Even if university students maintained >60 min/d of MVPA, poor sleep quality still led to a higher risk of depressive symptoms, with the same trend for both boys and girls (Table 4).

**Table 4:** Ordered logistic regression of the interaction between sleep quality and physical activity and depressive symptoms in university students in rural China.

Gender	Classification of interaction		Ordered logistic regression	
	PSQI	MVPA (min/d)	OR (95% CI)	*p-*value
Boys	Good	<30	1.22 (0.63 ~ 1.81)	<0.001
		30–60	2.94 (2.36 ~ 3.53)	<0.001
		>60	1.00 (Reference group)	
	Moderate	<30	2.8 (2.23 ~ 3.37)	<0.001
		30–60	0.9 (0.24 ~ 1.56)	<0.001
		>60	0.95 (0.04 ~ 1.85)	0.868
	Poor	<30	3.55 (2.99 ~ 4.12)	<0.001
		30–60	3.23 (2.64 ~ 3.82)	0.029
		>60	4.74 (3.89 ~ 5.58)	<0.001
Girls	Good	<30	1.79 (1.24 ~ 2.34)	<0.001
		30–60	1.38 (0.77 ~ 1.99)	<0.001
		>60	1.00 (Reference group)	
	Moderate	<30	2.11 (1.55 ~ 2.66)	<0.001
		30–60	1.11 (0.53 ~ 1.7)	<0.001
		>60	−0.1 (−1.24 ~ 1.05)	0.868
	Poor	<30	3.01 (2.46 ~ 3.56)	<0.001
		30–60	−2.27 (−4.31 ~ −0.24)	0.029
		>60	3.94 (2.88 ~ 5.00)	<0.001
Total	Good	<30	1.84 (1.45 ~ 2.24)	<0.001
		30–60	2.35 (1.94 ~ 2.76)	<0.001
		>60	1.00 (Reference group)	
	Moderate	<30	2.51 (2.12 ~ 2.91)	<0.001
		30–60	1.18 (0.75 ~ 1.61)	<0.001
		>60	0.52 (−0.18 ~ 1.22)	0.147
	Poor	<30	3.37 (2.98 ~ 3.76)	<0.001
		30–60	2.34 (1.91 ~ 2.77)	<0.001
		>60	4.40 (3.75 ~ 5.05)	<0.001

Age, father’s education, mother’s education, SES, and BMI were used as covariates in the ordered logistic regression. Sleep quality: Good ≤ 5, Moderate 6–7, Poor > 7. OR, odds ratio; 95% CI, confidence intervals. MVPA, Moderate-to-Vigorous Physical Activity; PSQI, Pittsburgh Sleep Quality Index.

## 4. Discussion

The results of this study showed that the detection rate of depressive symptoms in university students with sleep quality problems was significantly higher than that in university students without sleep quality. The higher the MVPA, the lower the detection rate of depressive symptoms in university students. The results of this study also showed that the rate of depressive symptoms among university students in rural areas of in Chinese was 33.3%, which was also higher among girls students (36.7%) than boys students (28.8%). This result was significantly higher than the findings for boys (6.7%) and girls (12.3%) in the United States (Chunnan et al., 2022). A study of depressive symptoms in adolescents confirmed that girls are influenced by their personality traits and prefer to close themselves off when they encounter problems, while boys prefer to express their stress or unhappiness through sports or by talking to others, resulting in a higher rate of depressive symptoms in girls than in boys (Shorey et al., 2022). In addition, the reasons for the differences in depressive symptoms may also be related to individual susceptibility and factors such as living environment and family environment (Mohan et al., 2017).

This study found a higher risk of depressive symptoms among university students in rural areas of China with poor sleep quality. A national study including 17,859 participants from 2007 to 2014 confirmed that both sleep duration and sleep difficulties (sleep problems and sleep disorders) were highly associated with the risk of depressive symptoms. And the study also investigated the combined association of sleep duration, sleep problems and sleep disorders with the risk of depressive symptoms, and the results also showed that participants with poorer overall sleep patterns were at higher risk of depressive symptoms (Chunnan et al., 2022). The strong association between sleep quality and depressive symptoms has been confirmed by studies (Hu et al., 2020). A longitudinal study showed that insomnia or biomarkers measured by polysomnography would affect mental health, leading to a 2.2–5.3-fold increase in the prevalence of depressive symptoms (Szklo-Coxe et al., 2010). In addition, a study of sleep interventions in depressed patients confirmed that improvements in sleep quality in depressed patients facilitated positive mood performance (Szklo-Coxe et al., 2010). A meta-analysis confirmed a “U” shaped relationship between sleep duration and the risk of depressive symptoms, suggesting that sleep duration is not the best, and that it is important to maintain a reasonable sleep duration and sleep quality (Zhai et al., 2015). Another study of Chinese participants also supports the “U” curve relationship between sleep duration and the risk of depressive symptoms (Sun et al., 2018). However, other studies do not support the conclusion that prolonged sleep is associated with an increased risk of depressive symptoms, possibly because of the different ages of the populations investigated (Jackowska and Poole, 2017). Another study of participants in rural China also showed (Jiang et al., 2020), Sleep quality showed a positive association with increased risk of depressive symptoms, which is consistent with our findings. A study on genetics confirmed that prolonged sleep >9 h/d and short sleep <7 h/d both increase the heritability of depressive symptoms (Watson et al., 2014).

Our findings showed that university students with MVPA of 30–60 min/d and < 30 min/d had a progressively higher risk of depressive symptoms compared to university students with MVPA >60 min/d. Several studies have found that maintaining a certain amount of physical activity time can reduce emotional symptoms and promote mental health. The possible mechanism is that exercise can promote the secretion of dopamine, 5-hydroxytryptamine, and brain-derived neurotrophic factor (BDNF) in the participants’ brains, which in turn can improve brain function through these neurotransmitters and play a role in regulating emotional symptoms (Wrann et al., 2013; Archer et al., 2014). A prospective meta-analysis reported that people with high levels of physical activity were 17% (95% CI, 12–21%) less likely to develop depressive symptoms compared to people with low levels of physical activity (Schuch et al., 2018). A study of 4,600 Irish adults confirmed that those who were physically active were less likely to develop depressive symptoms than those who were not physically active (McDowell et al., 2018). Another meta-analysis that included 49 studies confirmed that people with higher physical activity had lower odds of developing depressive symptoms compared to those with lower physical activity (OR = 0.83, 95% CI = 0.79, 0.88), and furthermore, physical activity had a protective effect on the occurrence of depressive symptoms in adolescents (OR = 0.90, 95% CI = 0.83, 0.98) (Schuch et al., 2018). The above results all confirm that maintaining a certain level of physical activity plays a positive role in preventing the occurrence of depressive symptoms. However, the factors leading to the occurrence of depressive symptoms are multifaceted and may also be related to the combined effects of other factors, which need to be further explored in the future.

In this study, the interaction analysis found that sleep quality and physical activity of university students in rural China interacted with the occurrence of depressive symptoms, which is consistent with the findings of related studies (Lovell et al., 2015). A study found that university students who participated in vigorous exercise had better sleep patterns and more slow-wave sleep, less light sleep as a percentage of total sleep, higher sleep quality, and a lower risk of psychological problems such as depressive symptoms than those who exercised lightly (Gerber et al., 2014). A 7 a cohort study found that the incidence of emotional instability was reduced in those who consistently participated in exercise and increased in those who had short sleep durations (Bowen et al., 2013). A large 15 a cohort follow-up study in Sweden found that the interaction between sleep and physical activity was a significant predictor of death, and that excessive sleep duration was associated with a high risk of death in people with low levels of physical activity (Bellavia et al., 2014). Another study also found that higher physical activity was effective in improving sleep quality while also reducing the occurrence of depressive symptoms, and that improved sleep quality was associated with lower serum cortisol levels and immune levels, which may also lead to a reduced risk of depressive symptoms (Passos et al., 2014). It has also been confirmed that higher physical activity is not better, and that the higher the amount of physical activity, the lower the additional potential health benefit and the greater the uncertainty, based on estimates of exposure prevalence in the included cohort, if less active adults met the current recommended physical activity recommendations, 11.5% (95% CI, 7.7% ~ 15.4%) of depressive symptoms cases could have been prevented (Pearce et al., 2022). Based on previous studies, this study confirmed the existence of interactions between sleep quality and physical activity with psychological symptoms among university students in rural areas of China, which provides a strong support for better promoting the mental health of university students in rural areas in the future.

This study has some strengths and limitations. Strengths: First, to the best of our knowledge, this is the first study to investigate the interactive effects of sleep quality and physical activity on depressive symptoms in university students in rural areas of China. Our study provides a reference and help to carry out prevention and intervention of depressive symptoms in university students in rural areas. Secondly, we conducted an extensive study in different rural areas of China, and the sample size has some advantages. However, our study also has some limitations. On the one hand, the study was a cross-sectional study, which could only confirm the association between sleep quality and physical activity and depressive symptoms, but could not understand the causal association that exists between them. Therefore, longitudinal studies that follow participants over time are necessary to determine causality. On the other hand, the study was conducted by questionnaire, and although the survey was anonymous, some students may conceal the truth, which may lead to bias in the results. For example, participants may have exaggerated or underestimated their physical activity levels or depressive symptoms. In addition, this study did not objectively measure sleep quality, but relied on self-reported measures, which could also lead to biases from reality. Future studies should consider using objective measures, such as actigraphy, to provide more accurate and reliable data. Fourth, this study did not take into account other covariates that may affect sleep quality, physical activity, such as stress levels, substance use, socioeconomic status, family background, or academic pressure etc., which could also have an effect on the it is necessary to include as many covariates as possible in future studies to accurately analyze the findings. Fifth, the study only focused on university students in rural areas of China, which limits the generalizability of the findings to other populations and settings. Future studies could compare the findings across different populations, such as urban vs. rural settings or different age groups, to provide a more comprehensive understanding of the relationship between sleep quality, physical activity, and depressive symptoms. Finally, this study only sampled 12 universities in three regions, which may not fully represent the actual situation of university students in rural China.

## 5. Conclusion

The incidence of depressive symptoms was higher in university students in rural areas of China, and there was an interaction between sleep quality and MVPA and depressive symptoms. The results confirm that even if university students maintain >60 min/d of MVPA time, if sleep quality is poor, it still leads to a higher risk of depressive symptoms, and the same trend exists for both boys and girls students. The evidence from this study suggests that maintaining a certain level of sleep quality is more important than maintaining higher physical activity. During future interventions, good quality of sleep should be guaranteed for university students, guaranteeing at least 8 h of sleep duration. Meanwhile, the duration of MVPA of not less than 60 min per day should be guaranteed to better promote the development of university students’ mental health.

## Data availability statement

The raw data supporting the conclusions of this article will be made available by the authors, without undue reservation.

## Ethics statement

The studies involving humans were approved by the Human Ethics Committee of Huanghuai University (201902-0219). The studies were conducted in accordance with the local legislation and institutional requirements. The participants provided their written informed consent to participate in this study.

## Author contributions

YY, HC, and ZZ: conceptualization. JL: methodology, supervision, and project administration. YY: investigation. YY and ZZ: data curation. YY and HC: writing – original draft and writing – review & editing. JL and HC: visualization. All authors contributed to the article and approved the submitted version.

## Conflict of interest

The authors declare that the research was conducted in the absence of any commercial or financial relationships that could be construed as a potential conflict of interest.

## Publisher’s note

All claims expressed in this article are solely those of the authors and do not necessarily represent those of their affiliated organizations, or those of the publisher, the editors and the reviewers. Any product that may be evaluated in this article, or claim that may be made by its manufacturer, is not guaranteed or endorsed by the publisher.
